# Supplementary material for: The causal relationship between the human gut microbiota and pyogenic arthritis: a Mendelian randomization study
Source: Front Cell Infect Microbiol. 2024 Nov 26;14:1452480. doi: 10.3389/fcimb.2024.1452480 (PMC11629706; doi:10.3389/fcimb.2024.1452480)
Supplement: Supplementary file 1 [file DataSheet1.zip › mendelian test/ebi-a-GCST90027566.csv_leave_one_out.pdf]

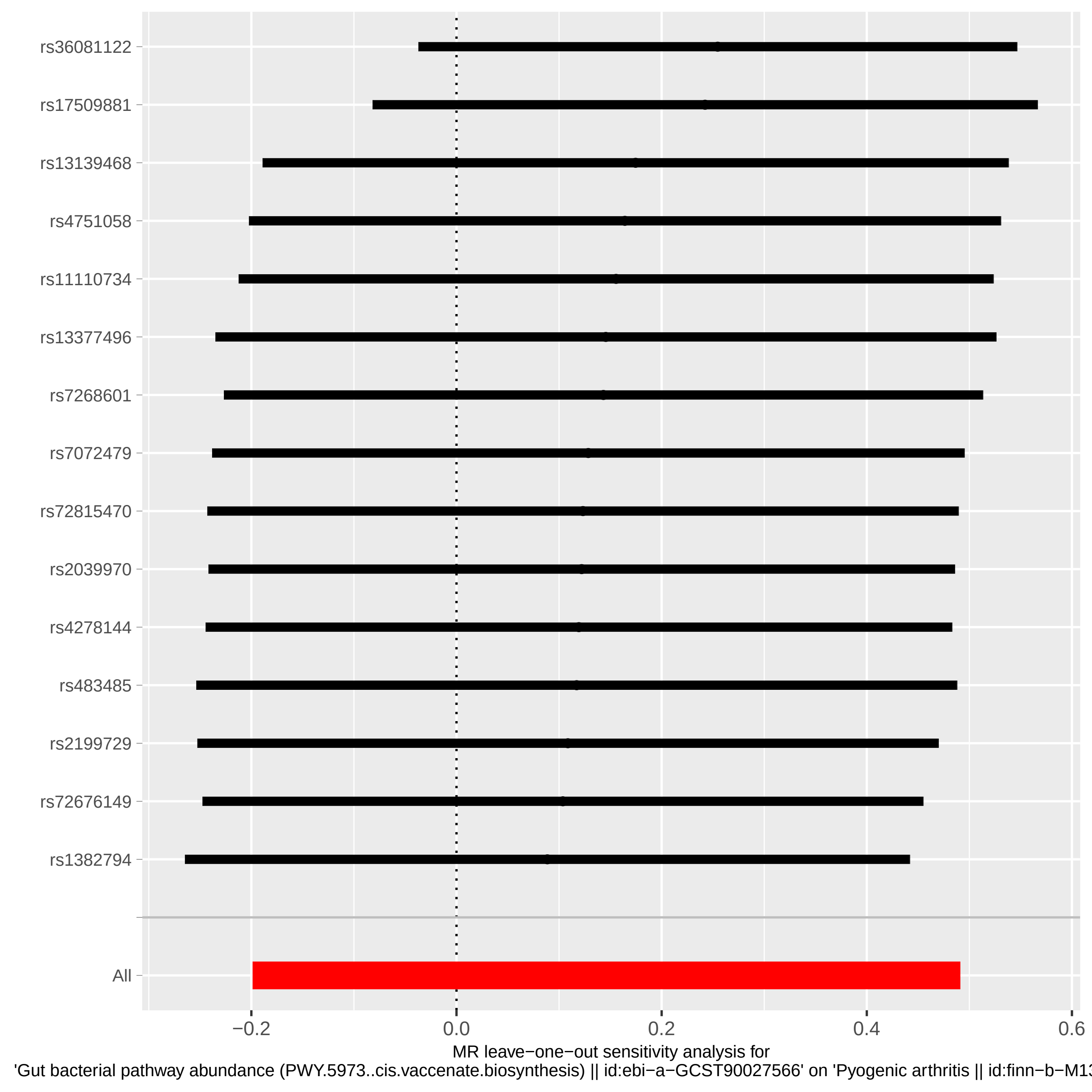
rs36081122
rs17509881
rs13139468
rs4751058
rs11110734
rs13377496
rs7268601
rs7072479
rs72815470
rs2039970
rs4278144
rs483485
rs2199729
rs72676149
rs1382794
All
−0.2
0.0
0.2
0.4
0.6
MR leave−one−out sensitivity analysis for
'Gut bacterial pathway abundance (PWY.5973..cis.vaccenate.biosynthesis) || id:ebi−a−GCST90027566' on 'Pyogenic arthritis || id:finn−b−M1
